# Endocrine disorders in patients with Fabry disease: insights from a reference centre prospective study

**DOI:** 10.1007/s12020-021-02918-4

**Published:** 2021-11-09

**Authors:** Christina Bothou, Felix Beuschlein, Albina Nowak

**Affiliations:** 1 Department of Endocrinology, Diabetology and Clinical Nutrition, University Hospital Zurich (USZ) and University of Zurich (UZH), Zürich, Switzerland; 2 Department of Internal Medicine, Psychiatry University Hospital Zurich, Zürich, Switzerland

**Keywords:** Fabry disease, Synacthen test, Thyroid-stimulating hormone, Vitamin D deficiency, Hypophosphatemia, Active renin

## Abstract

**Context:**

Fabry Disease (FD) is a rare X-linked storage disease characterised by a-galactosidase A deficiency and diffuse organ accumulation of glycosphingolipids. Enzyme replacement and chaperone therapies are only partially effective. It remains unclear if FD-related endocrine disorders contribute to the observed morbidity.

**Objective:**

To investigate the function of the endocrine system in patients with FD.

**Design:**

We conducted an observational prospective study from 2017 to 2020.

**Setting and patients:**

We included 77 patients with genetically confirmed FD (27 men, 20/27 Classic, 7/26 Late Onset phenotype, 50 women, 41/50 and 9/50 respectively), who are systematically followed by our reference centre.

**Results:**

36/77 (46.8%) patients had VitD deficiency (25(0H)VitD <20 μg/L) despite the fact that 19/36 (52.8%) were substituted with cholecalciferol. Only 21/77 (27.3%) patients had normal VitD levels without VitD substitution. 11/77 (14.3%) had significant hypophosphatemia (*p* < 0.80 mmol/L). Three new cases (3.9%) of subclinical, two (2.6%) of overt and six (7.8%) of known hypothyroidism were identified. Of note, men had significantly higher renin levels than women [61.4 (26.1–219.6) vs.25.4 (10.9–48.0) mU/L, *p* = 0.003]. There were no major abnormalities in adrenal, growth and sex-hormone axes. Patients of Classic phenotype had significantly higher High-Density Lipoprotein Cholesterol (HDL-C) levels (*p* = 0.002) and in men those levels were positively correlated with globotriaosylsphingosin (Lyso-Gb3) values. 10/77 (13%) of the patients were underweight.

**Conclusions:**

VitD supplementation should be considered for all patients with FD. Thyroid screening should be routinely performed. Malnutrition should be prevented or treated, particularly in Classic phenotype patients. Overall, our data suggest that FD specialists should actively seek and diagnose endocrine disorders in their patients.

## Introduction

Fabry Disease (FD) is a rare X- linked storage disease caused by mutations in the a-galactosidase A (*GLA*) gene leading to reduced activity of the encoded lysosomal enzyme (α-Galactosidase Α, α-Gal) [1]. As a consequence, globotriaosylceramide (Gb3) and other related glycosphingolipids are accumulated in the defective lysosomes of the patients who progressively develop diffuse organ damage [2].

The clinical phenotypes of the disease include the earlier Onset or Classic, and the attenuated, or so-called Late Onset phenotype. More specifically, in the Classic phenotype, males with little or no residual α-Gal A activity develop disease signs and symptoms already in childhood. These include acroparesthesias, angiokeratoma, corneal opacities, hypohidrosis and gastrointestinal symptoms. Progressively, accumulation of Gb3 and related glycoshingolipid metabolites in the different organs result in cardiac and renal complications as well as early strokes [3]. Males with the Late Onset phenotype have residual enzymatic activity and lack symptoms in childhood, they develop cardiac or, less frequently, renal manifestations usually later in adulthood. Females with FD are heterozygotes for the gene and may present with variable manifestations of FD, ranging from asymptomatic to severe, due to the skewed inactivation of the affected X-chromosome [4].

Since more than 20 years, treatment options for FD have become available and include intravenous enzyme replacement therapy (ERT) with agalsidase alfa or beta [2]. More recently, an oral pharmacological chaperone treatment (migalastat) became commercially available for patients with amenable mutations [5]. Moreover, clinical trials with substrate reduction [6] and gene therapy are currently ongoing [7]. However, the effectiveness of the treatment varies among patients and only ameliorates the symptoms or slows down the progression of the disease in many cases [8, 9, 10, 11]. Furthermore, many patients suffer from irreversible disease complications at treatment start, or, especially for those with the Late Onset phenotype, FD is diagnosed late in life because of the initially asymptomatic disease [12, 13].

Recently, the deacylated derivative of Gb3, globotriaosylsphingosine (Lyso-Gb3), has been identified as an important marker that can be measured in plasma and give insights into the disease severity [14, 15].

Regarding potential endocrine manifestations in patients with FD, the available data are sparse and conflicting. Endocrine organs are frequently referred as targets for glycosphingolipid accumulations [16, 17], basically, due to their high vascularisation and their low proliferation rate. Moreover, case–control studies regarding endocrine dysfunctions in patients with FD included only small number of patients [18, 19] or focused on a specific organ such as the pituitary [20] or thyroid gland [21]. Overall, the connection of endocrine diseases found in patients and the pathophysiology of FD remains unclear.

Therefore, we systematically analysed the mineral and the lipid metabolism, the weight status, the thyroid, adrenal, growth and sex-hormone axes in order to evaluate the need of systematic endocrine approach of patients with FD.

## Material and methods

### Patients and methods

Between July 2017 and December 2020, 77 patients with genetically confirmed FD visited the Outpatient Unit of the Endocrinology, Diabetology and Clinical Nutrition Clinic of the University Hospital of Zürich.

All patients were included in the study upon visit and written informed consent was obtained, according to the Institutional Instructions and in accordance with the Declaration of Helsinki of 1975 as revised in 2000. The central ethical Committee of the University of Zurich approved this study.

All patients had pathogenic *GLA* mutations. The pathogenicity and phenotyping of the mutations are shown in Supplementary Table 1 and were based on genotype and residual α‐Gal A activity in males and are published in the International Fabry Disease Genotype/Phenotype Database (www.dbFGP.org) and in previous studies [13, 15, 22]. Medical history was acquired, with focus on endocrine-related conditions. Fifty of the patients were asked an extra detailed fertility/pregnancy history. During a routine annual examination, we performed physical examination and assessed the patients clinically, biochemically, and hormonally.

Main clinical parameters, as well as main biochemical parameters including electrolytes, kidney function parameters and lipids, were measured.

Basal serum Thyroid-Stimulating Hormone (TSH), free T3 (fT3), free T4 (fT4), 25-hydroxy-VitaminD (25(OH)VitD), Follicle-stimulating hormone (FSH), Luteinizing Hormone (LH), Human Growth Hormone (HGH), Insulin-like growth factor 1 (IGF-I), total testosterone (total testo), free testosterone (free testo), Estradiol (E2), Aldosterone, active renin, Aldosterone to Renin Ratio (ARR), Dehydroepiandrosterone sulfate (DHEAS), Anti Mullerian Hormone (AMH), Sex Hormone Binding Globulin (SHBG), basal cortisol and Adrenocorticotropic hormone (ACTH) and stimulated Cortisol after short Synacthen test was assayed in FD patients. Regarding Synacthen test, serum cortisol was measured 60 min after intravenous injection of 250 μg ACTH. This dose had been chosen based on recent meta-analysis data which show that both high- and low-dose ACTH stimulation tests had similar diagnostic accuracy and both tests are adequate to rule in, but not rule out, secondary adrenal insufficiency [23], as well as known inconsistences in Synacthen dilution methods for low dose testing [24]. Prior evaluation of aldosterone and renin, ACE inhibitors and ARBs were paused for 5 days in all patients. All blood samples were obtained between 0800-h and 1000-h in the morning.

TSH, HGH, IGF-1, FSH, LH, SHBG, DHEAS, AMH, E2, total testo, fT4, fT3 and cortisol were measured by ElectroChemiLuminescence (ECL) Immunoassay with the use of the E170 autoanalyser (Roche, Basel, Switzerland). The coefficient of variations (CVs) of the methods used were 1.1% for TSH, 2.3% for HGH, 3.6% for IGF-1, 1.8% for FSH, 1.8% for LH, 1.5% for SHBG, 1.3–5,7% for DHEAS, 1.7% for AMH, 2% for E2, 2.5% for total testo, 2.3% for fT4 and 4.3% for fT3 and 3.4% for Cortisol. For ACTH, Aldosterone and Active Renin Chemiluminescence Enzyme Immunoassay (DiaSorin, Saluggia, Italy) (CVs 2.6–3.3%, 4.2–4.8% and 2.7–3.4%, respectively) with controls provided by the manufacturer was used and ARR was calculated as previously described [25]. 25(OH)VitD was measured by liquid chromatography tandem mass spectrometry (LC-MS/MS) with CV 5.7–7.1% and free testo by RadioImmunoAssay (RIA) (Diagnostic Products Corporation, Los Angeles, CA, USA) (CV 5.2–9.9%) with the use of the Immulite Autoanalyser (Siemens Diagnostic Healthcare). Free Androgen Index (FAI) = Total Testo (nmol/L)) × 100/Sex Hormone-Binding Globulin (SHBG) (nmol/ L), was calculated according to the above-mentioned formula and the normal values according to the literature for men was 30–150nmol/L.

All routine biochemical parameters and hormone profiles were determined in the Centre laboratory of the University Hospital of Zurich according to standard procedures.

Vitamin D (VitD) status was defined as deficiency when <20 μg/L and as insufficiency when <30 μg/L. The VitD supplementation status was drowned from the medical records in all patients. The serum VitD levels were analysed according to the season: summer (April to October) or winter (November to March). Due to circadian rhythm, the value of HGH was used only as an indication to exclude excess HGH production. Moreover, steroid hormones in female subjects were acquired on the day of the routine annual examination. Therefore, the presented normal values were adapted to include the different phases of the cycle and the case of each patient was evaluated individually.

Furthermore, levels of globotriaosylsphingosine (Lyso-Gb3) in the dried blood spots were measured by highly sensitive electrospray ionisation liquid chromatography tandem mass spectrometry (ESI LC-MS/MS) on a Shimadzu 8050 class I medical device (ARCHIMED Life Science GmbH, Vienna, Austria; www.archimedlife.com) as previously reported [26].

### Statistical analysis

Each individual patient was considered an independent case. For the analysis, we divided the cohort according to sex (male, female) and e phenotype (Classic, Late Onset). Moreover, for the analysis, we used the normal values provided by the Central laboratory of the University Hospital of Zurich, to estimate the number of patients that had normal values. Statistical analyses were performed using IBM SPSS Statistics for Windows (Released 2017, Version 25.0., IBM Corp, Armonk, NY). Graphs were generated using GraphPad Prism 5 (GraphPad Software, La Jolla, CA, United States). Variables were assessed for normality by visual evaluation of histograms and by Kolmogorov-Smirnov test. Categorical variables were expressed as proportions, continuous variables as medians with ranges. Differences between groups were assessed using Fisher’s exact test for categorical variables, Mann-Whitney *U* test for quantitative non-normally distributed variables and Student’s *t* test for normally distributed variables. Overall, comparisons of continuous variables between groups were carried out by using ANOVA or the Kruskal-Wallis test, as appropriate. Correlations were assessed by Pearson’s or Spearman’s correlation coefficient (*r*). A probability value of *p* < 0.05 was considered statistically significant for all tests, all testing was two-sided.

## Results

### Description of baseline characteristics of the patients

The patient group consisted of 27 men, 20 with the Classic and 7 with the Late Onset phenotype and 50 women, 41 of the Classic and 9 Late Onset phenotype.

The clinical history and the physical examination of all patients did not reveal any significant endocrine findings excluding fatigue, which is a frequently reported symptom and can be explained by multiple etiologies in the context of FD [27].

The demographics and baseline biochemical characteristics are depicted in Supplementary Table 2.

### Mineral metabolism

**Fig. 1:**
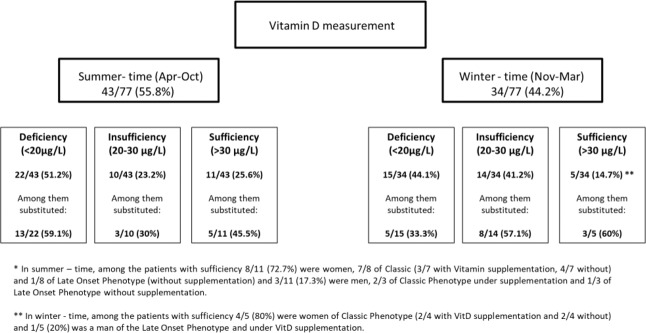
25-hydroxy-Vitamin D levels categorised as deficient, insufficient or sufficient in patients with FD under supplementation treatment or not, divided according to season

Regarding VitD, Ca and P status of the patients, values are shown in Table 1. VitD measurements were further analysed according to the season, as shown in Fig. 1. Interestingly, only few patients (16/77) (20.8%) had sufficient levels of VitD. More importantly, among male patients of Classic phenotype, only 1/20 (5%) had VitD levels within the normal range even in the summer time and following VitD supplementation. Of note, no correlation of the VitD status of the patients and the Lyso-Gb3 values was found (data not shown) and VitD levels were only slightly negatively correlated with eGFR for the subgroup of women with the Classic phenotype (Supplementary Figs. 2 and 3).

**Table 1:** Vitamin D and Phosphorus/Calcium values including substitution information.

		Men (*n* = 27)		Women (*n* = 50)		Normal values	*P*
	Phenotype	Classic (*n* = 20)	Late Onset (*n* = 7)	Classic (*n* = 41)	Late Onset (*n* = 9)		
**Mineral Metabolism Axis**	25 (OH)VitD (μg/L)	17 [14.15–26.15]	28 [21.65–31.8]	23.2 [17.9–30.8]	18.6 [16.8–27]	>20	0.062
	VitD supplementation dosage (IU/d)	1000 [1000–1200]	1000 [854.14–1250]	1000 [1000–1000]	-		0.479
	Phosphorus (mmol/L)	0.91 [0.76–0.97]	0.93 [0.88–0.97]	1.02 [0.91–1.1]	0.86 [0.79–0.96]	0.87–1.45	**0.003**
	Hypophosphatemia *n*, %	6(30)	1(14.3)	1(2.4)	3(33.3)	<0.80 mmol/L	
	Corrected Calcium total (mmol/L)	2.21 [2.16–2.24]	2.28 [2.2–2.36]	2.26[2.22–2.33]	2.27 [2.27–2.36]	2.19–2.54	0.7863
	Calcium Substitution *n*, %	3(15)	1(14.3)	3(7.3)	0(0)		

Continuous variables are presented as median and interquartile range, if more than two values were available; Kruksal-Wallis test was performed for the comparison of the groups; *p* < 0.05 was considered statistically significant; VitD, Cholecalciferol; 25(OH)VitD, 25-hydroxy-VitaminD

Interestingly, significant hypophosphatemia (<0.80) was detected in 11/77 (14.3%) of the patients. Four of them (4/11, 36.4%) were suffering from known secondary hyperparathyroidism due to VitD deficiency and were receiving substitution with cholecalciferol and calcium. Seven of them (7/11, 63.6%) had insufficiency or deficiency of VitD and only one patient (1/11, 9.1%) had hypophosphatemia of unclear aetiology.

No patients with calcium values (corrected for albumin levels) out of range were detected. Seven patients (7/77, 9.1%) with known secondary hyperparathyroidism and osteopenia in the context of Chronic Kidney Disease (CKD) were substituted with both cholecalciferol and calcium and one patient was on cinacalcet treatment.

### BMI status, lipid metabolism and glucose

Table 2 depicts the BMI status and lipid parameters. Regarding the lipid status, 10/77 (14%) patients had elevated levels of TC. In nine out of ten cases (90%) high TC was accompanied by high non- HDL-C and high LDL-C, 7/9 (77.8%) were overweight or obese and all of them (9/9, 100%) were under statin treatment and/ or dietary recommendations. One patient (10%) had elevated TC due to high HDL-C and was an underweight man of the Classic phenotype. Spearman’s correlation revealed that Lyso-Gb3 values are positively correlated with HDL-C (*r* = 0.283, *p* = 0.018). When restricted on individual subgroups, this correlation remained significant only for the subgroup of men with the Classic phenotype of the disease (*r* = 0.510, p = 0.026). Regarding the TGs, 9/77 (11.7%) patients had hypertriglyceridemia, 6/9 (66.7%) combined with the above-mentioned hypercholesterolaemia and 3/9 (33.3%) had isolated high TGs and all of them were obese. Only one patient with Classic phenotype (1/61, 1.6%) and diabetes mellitus Type 2 and one patient with familial MODY diabetes with Late Onset phenotype (1/16, 6.3%) showed an impaired glucose metabolism.

**Table 2:** Body Mass Index and Lipid Parameters of patients with FD

		Men (*n* = 27)		Women (*n* = 50)		Normal values	*P*
	Phenotype	Classic (*n* = 20)	Late Onset (*n* = 7)	Classic (*n* = 41)	Late Onset (*n* = 9)		
BMI status	BMI (kg/m^2^)	23.2 [20.6–25.1]	24.4 [21.9–26.6]	23.6 [20.8–26.5]	23.5 [22.1–27.6]	20–25	0.713
	<20 *n*, (%) 20–25 *n*, (%) 25–30 *n*, (%) >30 *n*, (%)	4 (20)	0 (0)	4 (9.75)	2 (22.2)		
		11 (55)	4 (57.1)	22 (53.65)	4 (44.4)		
		5 (25)	3 (42.9)	10 (24.4)	1 (11.1)		
		0 (0)	0 (0)	5 (12.2)	2 (22.2)		
Lipid status	TC (mmol/L)	4.7 [3.75–5.05]	3.8 [3.65–4.35]	4.5 [4.1–5.1]	4 [3.8–4.4]	<5.0	0.106
	High TC *n*, %	4 (20)	1 (14.3)	5 (12.2)	0 (0)		
	HDL-C (mmol/L)	1.5 [1.28–2.01]	1.07 [0.92–1.17]	1.6 [1.41–1.8]	1.31 [1.09–1.6]	>1.0	**0.002**
	Non-HDL-C (mmol/L)	2.8 [2.15–3.5]	2.7 [2.5–3.2]	2.8 [2.5–3.4]	2.8 [2.4–3.1]	<3.9	0.829
	LDL-C (mmol/L)	2.5 [1.7–2.95]	2.1 [1.9–2.6]	2.3 [1.9–3]	2.1 [1.8–2.6]	<3.0	0.734
	High LDL-C *n*, %	4 (20)	0 (0)	7 (17.1)	1 (11.1)		
	TG (mmol/L)	1.21 [0.7–1.44]	1.48 [1.28–1.73]	1.19 [0.75–1.45]	0.79 [0.72–0.85]	<2.0	0.086
	High TG *n*, %	2 (10)	1 (14.3)	5 (12.2)	1 (11.1)		

Continuous variables are presented as median and interquartile range, if more than two values were available; Kruksal-Wallis test was performed for the comparison of the groups; *p* < 0.05 was considered statistically significant; BMI, Body Mass Index; TC, Total Cholesterol; HDL-C, High-Density Lipoprotein Cholesterol; non-HDL-C, non-High-Density Lipoprotein Cholesterol; LDL-C, Low-Density Lipoprotein Cholesterol, TG; Triglycerides; n: number

### GH/IGF-1 axis

No abnormalities of the HGH/ IGF-1 axis were evident (Table 3). One patient (man, Classic phenotype) reported short statue with growth retardation in the past. However, previous results of two GHRH/Arginine tests showed no indication for growth hormone deficiency. Another patient (man, Classic phenotype) had a known hormone- inactive pituitary microadenoma. In this patient, acromegaly, among other endocrine activities, had been ruled out in the past by oral glucose tolerance test.

**Table 3:** GH/IGF-1 and thyroid of patients with FD

		Men (*n* = 27)		Women (*n* = 50)		Normal values	*P*
	Phenotype	Classic (*n* = 20)	Late Onset (*n* = 7)	Classic (*n* = 41)	Late Onset (*n* = 9)		
GH/IGF-1 Axis	HGH (μg/L)	0.8 [0.62–0.99]	2.44 [2.04–3.07]	0.37 [0.27–0.99]	0.31 [0.2–1]	<40	0.081
	IGF-1 (μg/L)	171.2 [117.8–200.6]	159.4 [143.4–207]	192.1 [136.3–244]	236.7 [174.7–266.9]	*age dependent	0.208
Thyroid Axis	TSH (mU/L)	2.41 [1.5–2.98]	1.84 [1.45–2.54]	2.11 [1.55–2.83]	1.53 [1.31–2.21]	0.16–4.25	0.462
	Elevated TSH *n*, %	2 (10)	0 (0)	2 (4.8)	1 (11.1)	>4.25 mU/L	
	Hypothyroidism under treatment *n*, %	0 (0)	1 (14.3)	5 (12.2)	0 (0)		
	fT4 (pmol/L)	15.3 [14.2–16.9]	17 [15.55–17.6]	15.2 [14.35–16.5]	15 [14.8–16.8]	m13.1–21.3 f 12.4–20.2	0.545
	fT3 (pmol/L)	4.9 [4.3–5.1]	4.7 [4.45–5.3]	4.85 [4.45–5.1]	5.1 [4.9–5.3]	m 4.1–6.7 f 3.6–6.4	0.530

Continuous variables are presented as median and interquartile range, if more than two values were available; Kruksal-Wallis test was performed for the comparison of the groups; *p* < 0.05 was considered statistically significant; TSH, Thyroid-Stimulating Hormone; fT4, free T4; fT3, free T3; HGH, Human Growth Hormone; IGF-1, Insulin-like growth factor 1.

### Thyroid axis

Six of 77 (7.8%) patients reported hypothyroidism in their medical history and were, at the time of the study, under levothyroxine substitution treatment resulting in euthyroidism. Regarding the aetiology, one patient reported hypothyroidism in the context of treatment with lithium. One patient with known Grave’s disease had been treated in the past with carbimazole and was euthyroid at the time of the current study.

TSH, fT4 and fT3 values of the patients did not differ among the subgroups (Table 3). Three patients (3/77, 3.9%) were found with high TSH values (>4.26 and <10.0 mU/L) with normal fT4 and fT3 and, due to high TSH in a repeated measurement after one month, they were defined as subclinical hypothyroid. Two Classic patients (2/77, 2.6%), one woman and one man were diagnosed with overt hypothyroidism for the first time and treatment with levothyroxine was initiated. Those patients were suffering from multiple Fabry-related morbidities and no direct link between referred unspecific symptoms such as fatigue and depression and hypothyroidism diagnosis could be found. The further evaluation of the aetiology of the above-mentioned cases of hypothyroidism is ongoing. No statistically significant correlation of the TSH level with the Lyso-Gb3 was detected (Supplementary materials Fig. 2) and TSH was only slightly negatively correlated with eGFR values (Supplementary materials Fig. 3) for men with the Classic phenotype. Finally, two patients (2/77, 2.6%) with repeatedly low fT3 and fT4 and normal TSH values were detected. However, none of them had increased Lyso-Gb3 values, severe nephropathy or acute illness or starving, a condition characterising these cases as non-thyroidal illness syndrome.

### Adrenal axis

All (77/77, 100%) patients had normal baseline ACTH and morning cortisol levels and unrestricted cortisol response upon short time Synacthen testing (62/62, 100%; Supplementary Fig. 1) and there was no clinical suspicion of adrenal insufficiency in any of the patients (Table 4). Of note, active renin levels in men were significantly higher than in women [61.4 (26.1–219.6) vs. 25.4 (10.9–48.0) mU/L, *p* = 0.003] and renin was positively correlated with Lyso-Gb3 (*r* = 0.282, *p* = 0.02) and Urea levels (*r* = 0.255, *p* = 0.036). No correlation between active renin levels and GFR or creatinine values was detected (data not shown).

**Table 4:** Adrenal Axis in patients with Fabry Disease

		Men (*n* = 27)		Women (*n* = 50)		Normal values	*P*
	Phenotype	Classic (*n* = 20)	Late Onset (*n* = 7)	Classic (*n* = 41)	Late Onset (*n* = 9)		
**Adrenal Axis**	ACTH (ng/mL)	12.5 [10–22]	16 [8.5–22]	10 [8–13]	7 [4–12.5]	0–60	**0.037**
	Basal Cortisol (nmol/L)	386.5 [250–369]	339 [153–477]	273.5 [206–363]	342 [185–305.5]	133–537	0.556
	Stimulated Cortisol (nmol/L)	655 [633–686]	738 [686–777]	686 [655–752]	688 [620–796]	>500	0.102
	Aldosterone (ng/L)	161 [83.8–205]	111 [84.1–146]	109 [69.2–165.5]	110.9 [50.6–285]	11.7–236	0.278
	Active Renin (mU/L)	58.4 [38.3–147.7]	104.2 [8.3–219.6]	27.2 [10.2–51.9]	18.7 [12.6–31.7]	2.8–39	**0.024**
	ARR (ng/mU)	3 [1.4–5.3]	1 [0.6–19.7]	6.2 [2.9–9.2]	6.1 [3.4–13.3]	<11.5	0.122
	DHEAS (μmol/L)						
	20–49 y, men	4.8 [3.5–6.3]	1.8			1.5–11	0.286
	>50 y, men	2.15 [1.25–3.25]	2.25 [1.25–3.25]			0.6–8.9	1.000
	20–49 y, women			3.2 [2.4–5.3]	4 [3.6–4.9]	1–7.9	0.363
	>50 y, women			1.5 [0.5–2.1]	0.2	0.6–8.9	0.154

Continuous variables are presented as median and interquartile range, if more than two values were available; Kruksal-Wallis test was performed for the comparison of the groups; *p* < 0.05 was considered statistically significant; ACTH, Adrenocorticotropic hormone; ARR, Aldosterone-to-Renin ratio

Overall, in the study group, no patient with known adrenal incidentaloma or known hormone-producing adenoma was present. Moreover, the medical records regarding medication and/or the presence of hypertension and hypokalaemia revealed no resistant hypertension in any of the subjects. Individual hormone parameters were evaluated in a case per case analysis. While in several patients various alterations of renin and/or aldosterone levels were detected, no suspicion of primary aldosteronism or zona glomerulosa insufficiency was present. No correlations between adrenal parameters and Lyso-Gb3 values or eGFR of the patients were evident (Supplementary materials Figs. 2 and 3, respectively).

### Reproductive history and steroid hormone profile of the male patients

Analytical fertility history acquired by 17 male patients (Table 6) showed that 9/17 (52.9%) of them acquired children. Those who did not fathered children were either of young age and/or did not wish to have children. Two/17 (5.9%) patients who reported delays in getting children, finally acquired children at latest after 2 years of efforts. SHBG beyond the normal range was detected in patients with reduced eGFR (Table 5). Total testosterone values, as well as gonadotropins, were within the normal range, if not affected by CKD (Table 5). No patient with clinical or biochemical suspicion of hypogonadismus was detected.

**Table 5:** Parameters of the gonadal axis in male patients according to phenotype

Gonadal Axis, Men				
Phenotype	Classic (*n* = 20)	Late Onset (*n* = 7)	Normal values	*p*
LH (IE/L)	5.1 [4.45–6.3]	6.5 [4.05–8.4]	1.5–12.4	0.731
FSH (IE/L)	4.15 [2.95–6.95]	5.7 [3.85–5.95]	1.7–8.6	0.406
E2 (pmol/L)	106 [91.5–141.5]	87 [8.3–107.5]	41–159	0.090
AMH (pmol/L)	39 [18.5–82]	30 [23–31]	5.5–103	0.156
Total Testo (nmol/L)				
20–49 y	20.3 [16.9–24.7]	11.6	8.64–29	0.133
>50 y	19.9 [13–26.75]	10.23 [5.65–14.35]	6.68–25.7	0.343
Free Testo (pmol/L)				
20–49 y	39.6 [34.8–112.3]	26.4	22.5–92.2	0.375
>50 y	31 [23.9–38]	19.85 [16.05–27.15]	7.6–73.9	0.190
FAI				
20–49 y	36.5 [36.5–46.8]	67.10	30–150	0.133
SHBG (nmol/L)				
20–49 y	51.9 [14–60.3]	67.10	18.3–54.1	0.133
>50 y	68.8 [48.7–98.8]	20.9 [16.4–49.5]	20.6–76.7	1.000

Continuous variables are presented as median and interquartile range, if more than two values were available; Mann-U Whitney test was performed for the comparison of the groups; *p* < 0.05 was considered statistically significant; LH, Luteinizing Hormone; FSH, Follicle-stimulating hormone; AMH, Anti Mullerian Hormone; FAI, Free Androgen Index; SHBG, Sex Hormone Binding Globulin; DHEAS, Dehydroepiandrosterone Sulfate; Total Testo, Total Testosterone; free Testo, free Testosterone; E2, Estradiol

**Table 6:** Fertility history. Infertility was defined as at least one year efforts to get pregnant without success

Fertiity History		
Parameter	Classic phenotype	Late Onset phenotype
Men	*n* = 13	*n* = 4
Children *n*, (%)	6 (46.2)	3 (75)
Infertility *n*, (%)	0 (0)	0 (0)
>1 year efforts to get children *n*, (%)	1 (7.6)	1 (25)
Mean number of children	2.5 [2,3]	2.5 [2,3]
Women	*n* = 28	*n* = 5
Children *n*, (%)	14 (50)	3 (60)
Infertility *n*, (%)	3 (10.7)	0 (0)
>1 year efforts to get pregnant *n*, (%)	3 (10.7) 1/3 via IVF	0 (0)
Miscarriages *n*, (%)	4(14.3)	1 (20)
Mean number of children	2 [2,6]	2.5 [2,3]
Mean age of menarche (years)	13 [12,14]	12 [12,12]
Mean age of menopause (years)	43 [42,44]	50 (1 patient)

Categorical variables were expressed as proportions and percentages and continuous variables as median and interquartile range, if more than two values were available; *n*: number

### Reproductive history and steroid hormone profile of the female patients

5/33 (15.2%) patients reported miscarriages in their past medical history (Table 6) but all of them delivered children. Three of 33 (9.1%) patients reported infertility and one out of those three had a history of primary infertility, which was substituted with oestrogen at the time of the study. Two of 33 patients (6.1%) achieved pregnancy only after consecutive efforts for more than a year and one following IVF. One/33 (3.0%) women had clinical and biochemical features of PCOS. No biochemical signs of hyperandrogenemia or FSH, LH, AMH and E2 dysregulation in other patients were detected (Table 7). Of note, none of the patients with the above-mentioned abnormalities was treated at the time of the study, or had been previously treated with migalastat. Based on beta HCG values, no pregnant women or patient with suspicion of an ovarian cancer was detected and HCG was lower than <0.3 U/L in all cases.

**Table 7:** Parameters of the gonadal axis in female patients according to phenotype

Gonadal Axis, Women				
Phenotype	Classic (*n* = 41)	Late Onset (*n* = 9)	Normal values	*p*
LH (IE/L)				
Before Menopause	5.25 [2.5–9.8]	2.3[1.25–4.15]	1–95.6	0.383
After Menopause	21.55[30.95–37.1]	22.7 [7.9–37.5]	7.7–58.5	0.526
FSH (IE/L)				
Before Menopause	5.05 [3.4–8.6]	3.4 [2.5–4.5]	1.7–21.5	0.530
After Menopause	60.9 [40.7–81.75]	47.55 [12.1–83]	25.8–134.8	0.526
E2 (pmol/L)				
Before Menopause	280 [197–562]	195 [148–386]	45–1461	0.144
After Menopause	2 [14–240]	75 [40–110]	<505	1.000
AMH (pmol/L)				
Before Menopause	3 [16–31]	2.45 [20–35]	*age dependent	0.367
After Menopause	<0.2	<0.2		
Total Testo (nmol/L)				
20–49 y	0.76 [0.41–1.04]	0.78 [0.61–1.28]	0.29–1.67	0.984
>50 y	0.52 [0.36–0.89]	1.65	0.10–1.42	0.462
Free Testo (pmol/L)				
20–49 y	4.6 [3.1–5.7]	5.2 [4.1–5.7]	2.8–15.6	0.796
>50 y	3.4 [2.9–4.6]	3.5	0.7–12.8	0.167
SHBG (nmol/L)				
20–49 y	74.1 [53.7–105]	74.8 [53.1–103]	32.4–128	0.619
>50 y	78.9 [61.1–118]	123	27.1–128	0.545

Continuous variables are presented as median and interquartile range, if more than two values were available; Mann-Whitney *U* test was performed for the comparison of the groups; *p* < 0.05 was considered statistically significant; LH, Luteinizing Hormone; FSH, Follicle-stimulating hormone; AMH, Anti Mullerian Hormone; DHEAS, Dehydroepiandrosterone Sulfate; Total Testo, Total Testosterone; free Testo, free Testosterone; E2, Estradiol

## Discussion

To our knowledge, this is the largest systematic endocrine evaluation of patients with FD. We took into account the whole genetic, clinical, and therapeutic information of the FD patients in association with the hormonal and biochemical parameters. Firstly, our results show that patients with FD are suffering from VitD deficiency and require VitD replacement, even during months with increased UV radiation. Secondly, 13% of the patients were underweight and no obesity was detected in the group of men of Classic phenotype. No diabetes mellitus was reported in men with Classic phenotype. Thirdly, hypothyroidism was detected in 14.3% of the patients. Fourthly, serum renin concentrations were increased in all male, while GH and Cortisol, as well as gonadal axes, were unremarkable.

VitD adequacy is of potential importance and should be carefully evaluated and substituted, considering, for example, that VitD deficiency has been correlated with worse prognosis of cardiomyopathy in FD [28]. In our analysis, VitD deficiency and insufficiency were commonly detected despite substitution with cholecalciferol. Of note, 55.8% of the measurements took place during summer months, when the UV exposure is expected to be higher [29]. However, in summer, only 25.6% of the patients had sufficient VitD and among them 45.5% were substituted. Furthermore, hypophosphatemia was demonstrated in many patients.

Possible explanations for the VitD deficiency include heat intolerance among patients with FD, which could result in sun avoidance [30]. Reduced sun exposure may also be a consequence of the multiple manifestations of the disease, including cardiac involvement leading to dyspnoea and reduced physical activity resulting in intense homing [31]. Additionally, Gb3 deposits in the skin, can interfere with the ViD synthesis [32–35]. Other explanations may include depression, frequently affecting FD patients, which can lead to non-compliance with medical treatment and, again, home staying [36]. In fact, multiple parameters may affect the nutritional and the VitD status of patients. Actively seeking for these parameters and adequate supplementation would improve the management of the patients and, in the long- term, their quality of life.

It is known that the risk of cardiovascular and cerebrovascular events is increased among patients with FD [11]. Lipid status, in most of our patients, was found within the normal range. Similarly, only a few elderly patients reported diabetes in their medical history. However, the cardiac and renal manifestations of FD, such as left ventricle hypertrophy, cardiomyopathy, and CKD, increase independently the risk for cardiovascular events in those patients and no suspicion of high prevalence of metabolic co-factors has been reported in the literature [37]. Similarly, high incidence of strokes among patients with FD is related to cerebrovascular involvement and the Gb3 and Lyso-Gb3 accumulation [38]. Pointing to the same direction, in our study, no correlation between Lyso-Gb3 and TC, non-HDL-C, LDL-C and TG was detected, and their levels did not differ significantly among the different disease phenotypes. Few patients in our cohort with elevated TC or TG levels carried additional metabolic risk factors, such as obesity, and this could explain their lipid profile. Interestingly, we reported a positive correlation between HDL-C levels and plasma Lyso-Gb3 values, especially for the subgroup of men with the Classic phenotype. Our finding is in accordance with the previous study from *Stepien* et al., who detected remarkably elevated HDL-C in a cohort of 72 adults with FD. In the same study, HDL-C levels and the overall lipid profiles did not change significantly despite enzyme replacement therapy in the long- term [39]. Additionally, Cartwright et al. also presented high HDL-C, normal LDL-C and TG and slightly increased total cholesterol in FD patients [40]. A possible explanation is that the physiology of lipoproteins is affected by glycosphingolipid accumulation in the endothelial cells leading to an inhibition of apoA-mediated cholesterol efflux. Thus, HDL-C increase may be related to the pathophysiology of the FD, and a protective effect of HDL-C could explain why FD patients rarely develop coronary artery disease [11, 37].

A significant number of patients were underweight. Glycosphingolipids are known to be accumulated in the gastrointestinal system. In a study, 70% of FD males reported gastroenterological symptoms at least on a weekly basis and 47% considered themselves underweight, as a consequence of the abdomen symptomatology [41]. Hence, recent data support that enzyme replacement therapy can alleviate gastroenterological problems and results in increase in body weight [42]. However, the effect of the gastroenterological manifestations on the nutrition status and the adequacy of vitamins, minerals, and micronutrients in patients with FD is still under investigation. Aguilera-Correa et al. demonstrated that Lyso-Gb3 accumulation in colon affects gut microbiota [43]. Overall, patients with FD often suffer from cachexia while little is known about the dietary status of FD patients. On one hand, cachexia can be caused by systemic inflammation; on the other hand, nutrient absorption can be impaired by sphingolipid accumulation, in gastrointestinal autonomous nervous system. Malnutrition should be managed by nutritional support with, for example, high caloric drinks and nutritional counselling.

As mentioned above, the GH/IGF-1 axis of the patients in our study was – based on baseline endocrine parameters - unaffected. Thus, no further systematic assessment of this axis seems to be required, unless a clinical suspicion is present. Maione et at. have studied the pituitary function in 28 patients with FD and respective controls, by assessing MRI, baseline hormonal and stimulated hormonal parameters [20]. Even though the authors found an increase incidence of empty sella in patients with FD, pituitary function was not impaired. Due to correlations of pituitary size and disease progression, they suggest frequent pituitary work up in FD patients. In other studies, pituitary hormones were intact [18]. Overall, to avoid possible hormonal dysregulation in the context of empty sella syndrome [44], frequent pituitary workup is suggested in patient with FD and related clinical suspicion or empty sella findings in MRI.

Previous studies have shown increased incidence of subclinical hypothyroidism among patients with FD. More specifically, a study that included 11 patients with FD and their controls reported subclinical hypothyroidism in 4 cases [21]. However, half of the patients had also positive anti-thyroid antibodies. Increased prevalence of subclinical hypothyroidism in combination with hypoechoic findings in thyroid ultrasound (US) without a presence of thyroid antibodies was also reported by other studies [18]. Moreover, a later study from the same group investigated in more detail thyroid function before and after ERT and reported that subclinical hypothyroidism and related US alterations, improved following intervention [45]. Among our patients, we found a constellation subclinical hypothyroidism in 3.9% and clinical hypothyroidism in 2.6%. Further 7.8% of the patients had known hypothyroidism (83.3% of unknown aetiologies and one case, 16.7% due to regular lithium intake) and were substituted with levothyroxine. Sonographic or antibody information was not available as their systematic measurement is not part of clinical routine in FD patients. TSH values were unrelated to eGFR or Lyso-Gb3 values. Pathophysiologically, it has been shown previously that Gb3 may accumulate in the thyroid gland of FD patients [17], which could also be the basis of mild hypothyreoidism in many of our patients. If these accumulations are adequate or necessary event to stimulate disease-related thyroid dysfunction and by which mechanism (e.g. autoimmune), needs to be further studied. Overall, our findings indicate, in accordance with previous studies, that subclinical hypothyroidism is common among patients with FD and thyroid function should be monitored by the treating physician.

Interestingly, renin levels were found to be elevated in men of both Classic and Late Onset phenotype, which was positively correlated with Lyso-Gb3 levels. Because Classic phenotype primarily affects vascular endothelial cells [46], endothelial dysfunction can lead to an impaired renal microcirculation resulting in increased active renin levels. In addition, CKD caused by the Fabry-nephropathy itself and the cardio-renal syndrome [11] can lead to reduced renal blood circulation also resulting in increased renin levels. By RAAS activation, renin can lead to arterial hypertension [47], progressing nephropathy [48], cardiovascular remodelling [49] and, therefore, accelerate cardiovascular events. Thus, it is conceivable that RAAS blocker should be a part of antihypertensive therapies of FD patients.

Cortisol response to ACTH stimulation was appropriate in all the patients of our cohort. Previous studies reported insufficient adrenal response in dynamic tests but no case with adrenal insufficiency was confirmed [18].

Male patients with FD have been previously reported to suffer from azoospermia and experience fertility issues [18]. Testicular and epidydimal involvement was also detected in a case report of a male patient [16]. However, other contradictory data show unimpaired testicular function in 11 male patients with FD [19]. In our study, despite the expected impairment of the SHBG and LH levels in patients with CKD, no suspicion of hypogonadismus was detected [50].

Regarding the fertility of the female patients, many patients reported miscarriages, in accordance with previous studies [18, 19]. However, all of them had children. For the reported cases of infertility, further investigation is needed to assess the disease- contribution to that. Of note, migalastat had been reported to impaired fertility in animal models with FD [5]. In our cohort no such correlation was found.

Limitations of our study include the lack of a control group and, partly, missing information regarding the aetiology (autoimmune, by Gb3 depositions or other) of the subclinical and overt hypothyroidism cases and the infertility of the female patients. However, the normal values of the Tertiary Centre, as revised in a systematic basis according to the latest guidelines [51], were considered as adequate for the current study. Moreover, for the complete evaluation of the pituitary and the male fertility, MRI imaging and semen analysis, respectively, would be needed but have not been feasible in the context of the current study design.

The strengths of our study include the availability of an extensive and recent dataset reflecting the current endocrine status of the patients with genetically confirmed FD that belong to a large cohort and are systematically followed by a reference centre.

In conclusion, the clinical implications of our study are, firstly, that VitD supplementation should be considered for all patients with FD, even in summer. Secondly, a thyroid screening should be regularly performed, for example during annual examinations, in order to timely diagnose overt hypothyroidism. Thirdly, malnutrition should be treated or prevented, particularly in Classic phenotype patients, using high caloric food and supplements, as tolerated due to gastrointestinal disturbances, and supported by nutritional counselling. Overall, these data provide a reassuring ground for suggesting to the FD specialist actively seek and diagnose endocrine disorders in their patients with the goal to optimise their health care.

## Supplementary Information

Supplementary materials

## Data Availability

Upon request by the reviewers.
