# Supplementary material for: Endocrine disorders in patients with Fabry disease: insights from a reference centre prospective study
Source: Endocrine. 2021 Nov 9;75(3):728–39. doi: 10.1007/s12020-021-02918-4 (PMC8888367; doi:10.1007/s12020-021-02918-4)
Supplement: Supplementary file 1 — Supplementary materials [file 12020_2021_2918_MOESM1_ESM.docx]

**Endocrine Disorders in Patients with Fabry disease: Insights from a reference centre prospective study.**

**Christina Bothou^1^, Felix Beuschlein^1^, Albina Nowak^1,2^**

^1^ Department of Endocrinology, Diabetology and Clinical Nutrition, University Hospital Zurich (USZ) and University of Zurich (UZH), Zürich, Switzerland

^2^ Department of Internal Medicine, Psychiatry University Hospital Zurich, Zürich, Switzerland

**Supplementary Table 1: Genetic characteristics of the Fabry patients included in the study.**

| Age | GLA Mutation | Predicted enzyme protein change |
| --- | --- | --- |
| Classic  Phenotype, men (n=20) |  |  |
| 57 | Deletion exon 2  (g2962, g5871) |  |
| 41 | c.125T>C | p.M42T |
| 42 | c.136C>T | p.H46Y |
| 49 | c.370-2A>G  (IVS2-2A>G) | Functional null allele due to splice site mutation |
| 41 | IVS3+405T>G | Functional null allele due to splice site mutation |
| 30 | c.559_560delAT | p.Met187Valfs*6 |
| 59 | c.581C>T | p.T194I |
| 73 | c.581C>T | p.T194I |
| 57 | c.581C>T | p.T194I |
| 41 | c.581C>T | p.T194I |
| 34 | c.679C>T | p.R227X |
| 22 | c.744_745delTA | p.F248L |
| 20 | c.796G>T | p.D266Y |
| 45 | c.827G>A | p.S276N |
| 59 | c.899T>A | p.L300H |
| 45 | c.1033T>C | p.S345P |
| 46 | c.1033T>C | p.S345P |
| 32 | c.1055_1057dupCTA | p.A352_M353insT |
| 22 | c.1235_1236delCT | p.T412SfsX38 |
| 50 | c.1235_1236delCT | p.T412SfsX38 |
| Classic Phenotype, women (n=41) |  |  |
| 53 | c.72G>A | p.W24X |
| 18 | Deletion exon 2  (g2962, g5871) |  |
| 32 | c.125T>C | p.M42T |
| 41 | c.125T>C | p.M42T |
| 29 | c.125T>C | p.M42T |
| 52 | c.365delA | p.N122IfsX |
| 71 | c.370-2A>G | Functional null allele due to splice site mutation |
| 68 | c.514T>C | p.C172R |
| 38 | c.581C>T | p.T194I |
| 70 | c.581C>T | p.T194I |
| 68 | c.581C>T | p.T194I |
| 42 | c.581C>T | p.T194I |
| 40 | c.581C>T | p.T194I |
| 26 | c.581C>T | p.T194I |
| 28 | c.581C>T | p.T194I |
| 46 | c.581C>T | p.T194I |
| 44 | c.581C>T | p.T194I |
| 71 | c.581C>T | p.T194I |
| 69 | c.581C>T | p.T194I |
| 47 | c.640-3C>G | Functional null allele due to splice site mutation |
| 55 | c.680G>A | p.R227Q |
| 33 | c.704C>A | p.S235Y |
| 62 | c.796G>T | p.D266Y |
| 60 | c.796G>T | p.D266Y |
| 30 | c.899T>A | p.L300H |
| 29 | c.901C>T | p.R301X |
| 40 | c.901C>T | p.R301X |
| 28 | c.950T>C | p.I317T |
| 47 | c.1033T>C | p.S345P |
| 21 | c.1033T>C | p.S345P |
| 28 | c.1033T>C | p.S345P |
| 55 | c.1033T>C | p.S345P |
| 32 | c.1033T>C | p.S345P |
| 35 | c.1055_1057dupCTA | p.A352_M353insT |
| 37 | c.1167dupT | p.V390CfsX9 |
| 42 | c.1167dupT | p.V390CfsX9 |
| 58 | c.1167dupT | p.V390CfsX9 |
| 21 | c.1167dupT | p.V390CfsX9 |
| 74 | c.1167dupT | p.V390CfsX9 |
| 44 | c.1235_1236delCT | p.T412SfsX38 |
| 68 | c.1235_1236delCT | p.T412SfsX38 |
| Late Onset Phenotype,  men (n=7) |  |  |
| 63 | c.337T>C | p.F113L |
| 46 | c.337T>C | p.F113L |
| 51 | c.613C>T | p.P205S |
| 67 | c.644A>G | p.N215S |
| 66 | c.713G>A | p.S238N |
| 50 | c.902G>A | p.R301Q |
| 43 | c.902G>A | p.R301Q |
| Late Onset Phenotype, women (n=9) |  |  |
| 40 | c.337T>C | p.F113L |
| 39 | c.337T>C | p.F113L |
| 35 | c.337T>C | p.F113L |
| 29 | c.337T>C | p.F113L |
| 18 | c.337T>C | p.F113L |
| 37 | c.337T>C | p.F113L |
| 16 | c.337T>C | p.F113L |
| 42 | c.902G>A | p.R301Q |
| 79 | c.902G>A | p.R301Q |

**Supplementary Table 2: Baseline clinical and biochemical characteristics.** Continuous variables are presented as median and interquartile range, if more than two values were available; Kruksal-Wallis test was performed for the comparison of the groups; p<0.05 was considered statistically significant; eGFR, Estimated Glomerular Filtration Rate; ALT, alanine aminotransferase; NT-Pro-BNP, N-terminal Pro Brain Natriuretic Peptide; Lyso-Gb3, Globotriaosylsphingosine; ERT, Enzyme- Replacement Therapy; Crea, Creatinine.

|  | **Men (n=27)** | | **Women (n=50)** | | **Normal values** | **P** |
| --- | --- | --- | --- | --- | --- | --- |
| **Phenotype** | **Classic (n=20)** | **Late Onset (n=7)** | **Classic (n=41)** | **Late Onset (n=9)** |  |  |
| Age | 44 [32,53.5] | 51 [48,64.5] | 43 [32,58] | 37[29,40] |  | 0.083 |
| Plasma Lyso-Gb3 (ng/mL) | 36.8 [27.5, 42.35] | 7.6 [5.45,8.85] | 9 [6.4,11.1] | 2.4 [2.3,3.2] | cut-off < 1.8 | **<0.001** |
| Years in ERT Treatment | 17.5 [9,19] | 10 [7.5,10.5] | 11 [7,14] | - |  | **0.039** |
| Years on chaperone Treatment | - | 2.65 [1.3,4] | 3 [2.75,3.25] | 4 |  | **0.677** |
| Sodium (Na) (mmol/L) | 139 [138,140] | 139 [138,141] | 139 [138,140] | 139 [138,141] | 136- 145 | 0.881 |
| Potassium (K) (mmol/L) | 4.1 [3.9,4.35] | 4.2 [4.0,4.4] | 3.8 [3.6,4] | 3.8 [3.7,4.1] | 3.3- 4.5 | **0.007** |
| Bicarbonates (mmol/L) | 25 [23,25] | 26 [24.5,26.5] | 24 [22,25] | 22 [21,23] | 22- 29 | **0.013** |
| eGFR (ml/min/1.73m^2^) | 102 [71,116] | 74 [66.5,86.5] | 91 [84,113] | 111 [96.5,120.5] |  | 0.118 |
| Urea (mmol/L) | 4.2 [3.4,5.6] | 5.7 [5.45,7.35] | 4.1 [3.4,4.85] | 4.1 [3.6,4.9] | 2.86- 8.21 | 0.075 |
| Crea (μmol/L) | 78 [72,105] | 102 [87,107] | 66 [58,75] | 59 [57,66] | 44- 80 | <0.001 |
| Urine Crea (mmol/L) | 5.71 [3.43,7.54] | 8.67 [6.72,9.82] | 3.84 [2.36,6.01] | 10.77 [7.31,16.92] | 2.5- 19.2 | **0.001** |
| ATL (U/L) | 24.5 [20,38] | 30 [19.5,46.5] | 17 [15,23] | 20 [16,23] | <35 | **0.004** |
| Random Glucose (mmol/L) | 4.8 [4.6-5.7] | 5.7 [5.25-6.6] | 5.1 [4.8-5.5] | 4.8 [4.6-5.1] | <11 | 0.129 |
| Troponin T (ng/L) | 9 [5.5,35.5] | 33 [26,71] | 8 [4,32] | 3 [1,6] | <14 | **0.006** |
| NT-Pro-BNP (ng/L) | 62 [23.5,423.5] | 132 [106,267] | 69 [46,472] | 61 [44,153] | <301 * | 0.345 |

**Figures**

**Supplementary Figure 1: Basal and stimulated cortisol levels in patients with FD, according to sex and phenotype.**


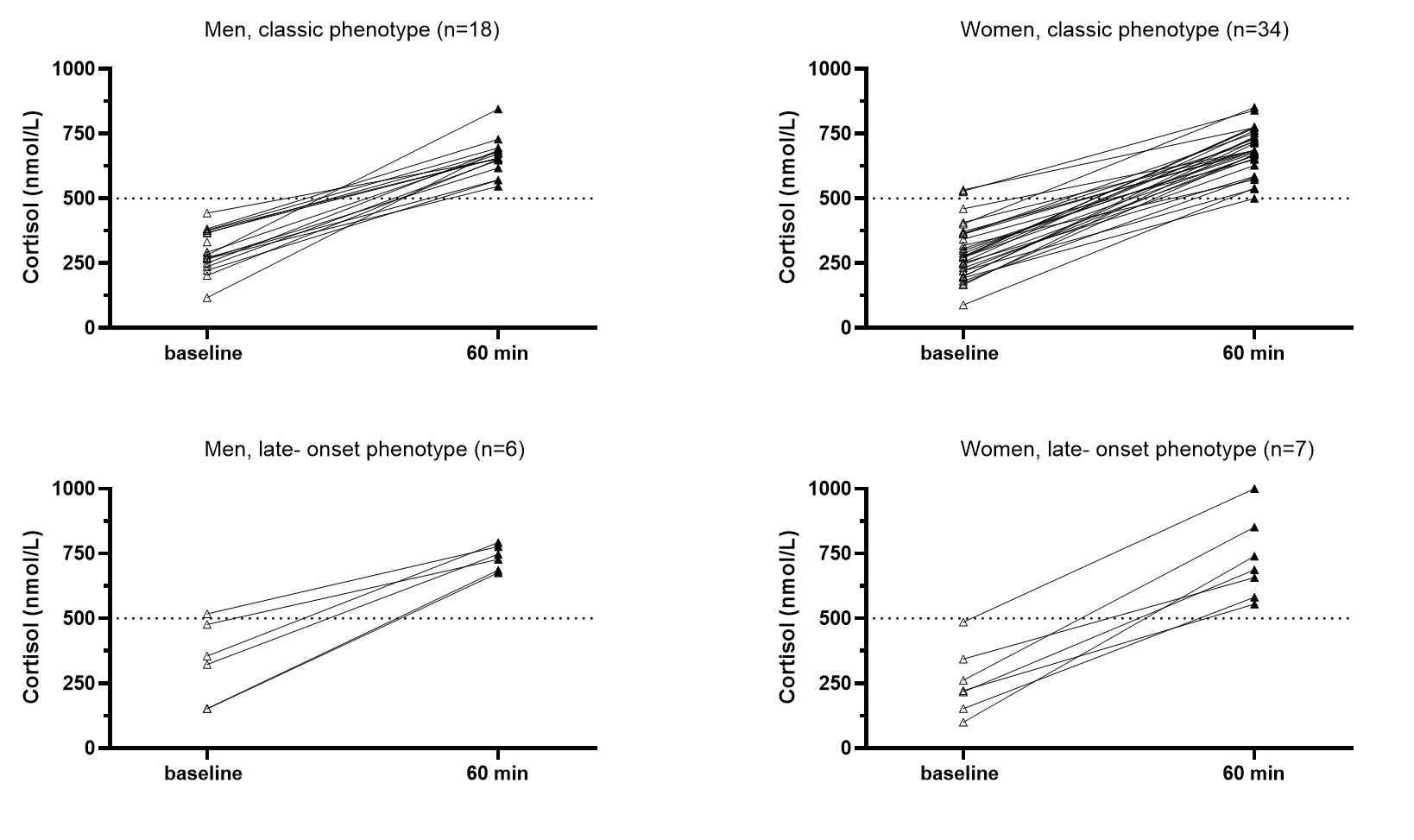


FD, Fabry Disease

**Supplementary Figure 2: Relation between Lyso-Gb3 levels of male and female patients with Classic FD and TSH, ACTH, stimulated cortisol and 25(OH)VitD values.**


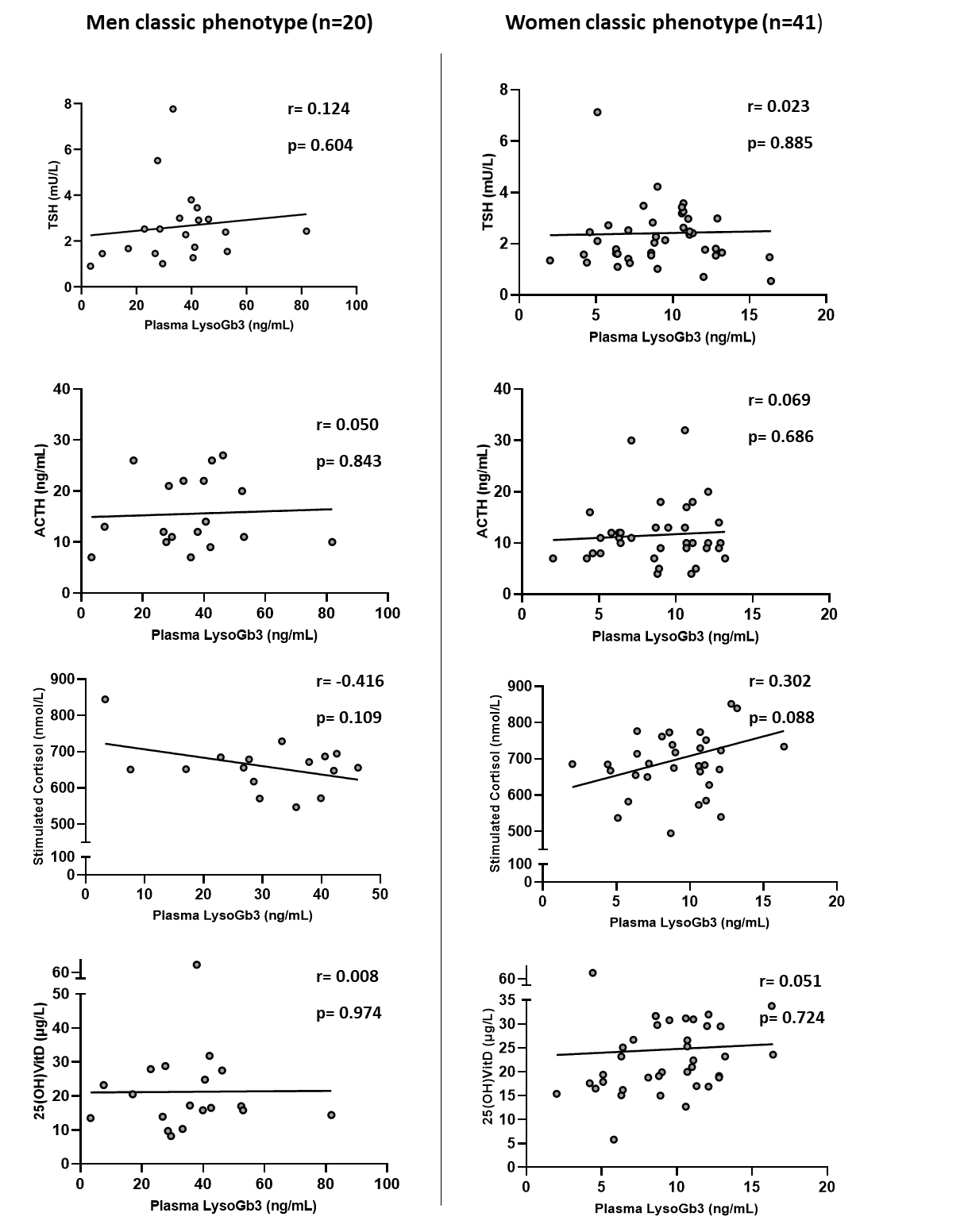


FD, Fabry Disease; 25(OH)VitD, 25-hydroxy-Vitamin D; Lyso-Gb3, Globotriaosylsphingosine.

TSH, Thyroid-Stimulating Hormone; ACTH, Adrenocorticotropic hormone;

**Supplementary Figure 3: Relation between eGFR levels of male and female patients with Classic FD and TSH, ACTH, stimulated cortisol and 25(OH)VitD values.**

**
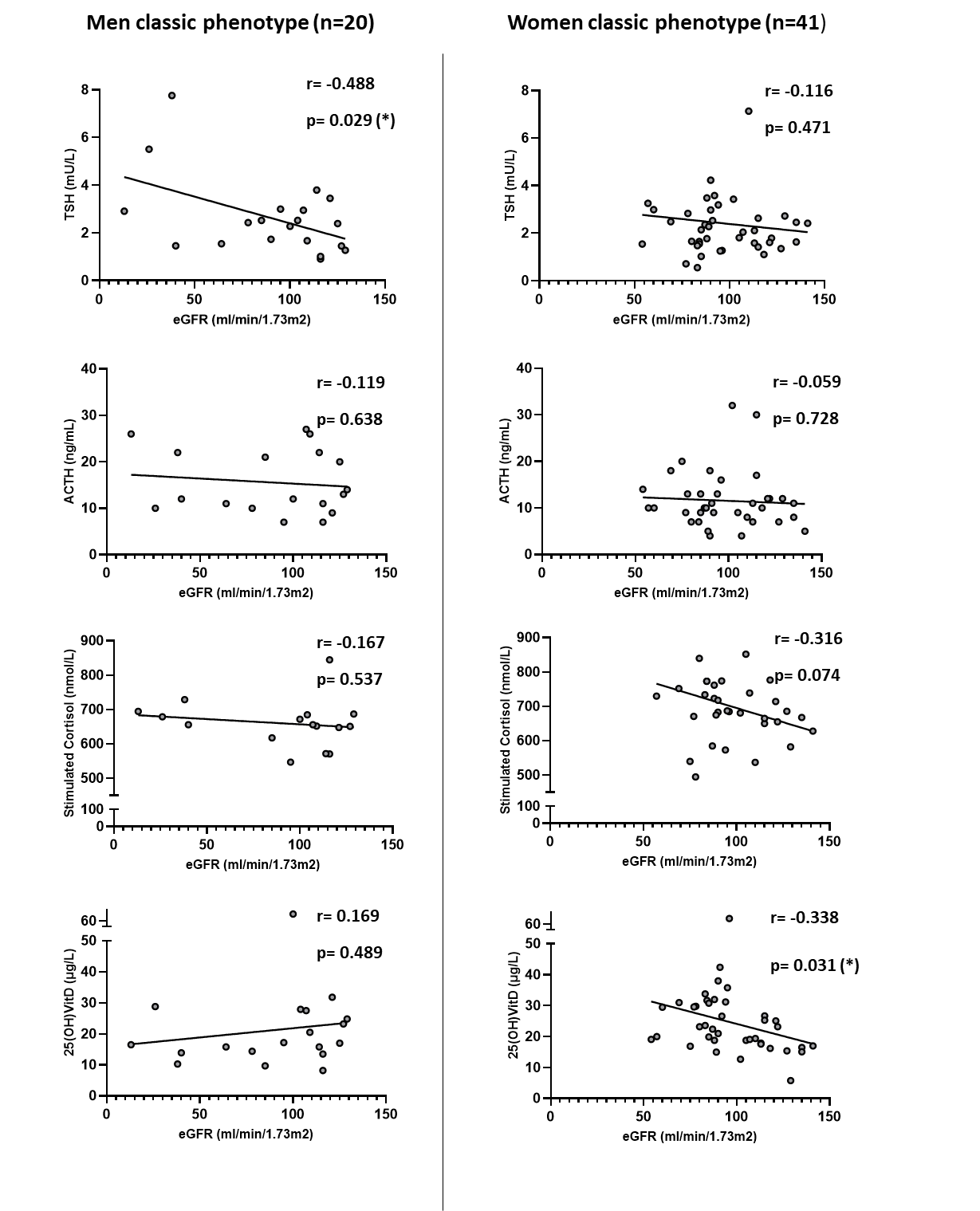
**

FD, Fabry Disease; 25(OH)VitD, 25-hydroxy-Vitamin D; eGFR, Estimated Glomerular Filtration Rate; TSH, Thyroid-Stimulating Hormone; ACTH, Adrenocorticotropic hormone.
